# Enhanced Regeneration of Vascularized Adipose Tissue with Dual 3D-Printed Elastic Polymer/dECM Hydrogel Complex

**DOI:** 10.3390/ijms22062886

**Published:** 2021-03-12

**Authors:** Soojin Lee, Hyun Su Lee, Justin J. Chung, Soo Hyun Kim, Jong Woong Park, Kangwon Lee, Youngmee Jung

**Affiliations:** 1 Center for Biomaterials, Biomedical Research Institute, Korea Institute of Science and Technology, Seoul 02792, Korea; dltnwls830@snu.ac.kr (S.L.); chungjj@kist.re.kr (J.J.C.); soohkim@kist.re.kr (S.H.K.); 2 Program in Nanoscience and Technology, Graduate School of Convergence Science and Technology, Seoul National University, Seoul 08826, Korea; hyun118soo@snu.ac.kr; 3 NBIT, KU-KIST Graduate School of Converging Science and Technology, Korea University, Seoul 02841, Korea; 4 Department of Orthopedic Surgery, Korea University Anam Hospital, Seoul 02841, Korea; ospark@korea.ac.kr; 5 Department of Applied Bioengineering, Graduate School of Convergence Science and Technology, Seoul National University, Seoul 08826, Korea; 6 School of Electrical and Electronic Engineering, YU-KIST Institute, Yonsei University, Seoul 03722, Korea

**Keywords:** 3d printing, PLCL, decellularization, angiogenesis, dECM hydrogel, adipose tissue regeneration

## Abstract

A flexible and bioactive scaffold for adipose tissue engineering was fabricated and evaluated by dual nozzle three-dimensional printing. A highly elastic poly (L-lactide-co-ε-caprolactone) (PLCL) copolymer, which acted as the main scaffolding, and human adipose tissue derived decellularized extracellular matrix (dECM) hydrogels were used as the printing inks to form the scaffolds. To prepare the three-dimensional (3D) scaffolds, the PLCL co-polymer was printed with a hot melting extruder system while retaining its physical character, similar to adipose tissue, which is beneficial for regeneration. Moreover, to promote adipogenic differentiation and angiogenesis, adipose tissue-derived dECM was used. To optimize the printability of the hydrogel inks, a mixture of collagen type I and dECM hydrogels was used. Furthermore, we examined the adipose tissue formation and angiogenesis of the PLCL/dECM complex scaffold. From in vivo experiments, it was observed that the matured adipose-like tissue structures were abundant, and the number of matured capillaries was remarkably higher in the hydrogel–PLCL group than in the PLCL-only group. Moreover, a higher expression of M2 macrophages, which are known to be involved in the remodeling and regeneration of tissues, was detected in the hydrogel–PLCL group by immunofluorescence analysis. Based on these results, we suggest that our PLCL/dECM fabricated by a dual 3D printing system will be useful for the treatment of large volume fat tissue regeneration.

## 1. Introduction

Development of enhanced vascularized adipose tissue is a prospective issue for not only adipose tissue but also soft tissue engineering in modern healthcare [1,2,3,4]. Adipose tissue engineering has been studied and is required in soft tissue deficiency, such as traumatic injuries, extensive burn defects, innate defects, and defects after cancer treatments. Most of all, as the number of patients diagnosed with breast cancer increases, mammotomies or lumpectomies are performed more often. Therefore, interest has increased in breast regeneration to improve the quality of life of cancer patients. Adipose tissue is the largest and ubiquitous tissue in the body; thus, unlike other organs or tissues, it can easily be acquired [5,6,7]. Although autologous fat grafts have been regarded as an ideal way to augment soft tissue lost because of fewer immunological rejections and complications, their problem is that the engrafted fat has a poor volume maintenance rate after surgery, with a 40–60% volume loss because of ineffective integration with the mature adipose tissue [8,9,10,11]. In the case of small defects, it may be efficient; however, not for large defects.

Various trials for adipose tissue regeneration have been conducted [10]. Many studies with diverse materials have been performed to replace autografting, but the results were not satisfactory [12,13]. Tissue engineering with scaffolds has potential for developing and reconstructing adipose tissue. Scaffolds are one of fundamental components [11,14], which contribute significantly to tissue engineering. A scaffold primarily supports a three-dimensional (3D) structure implanted into host tissue [8]. To fabricate a 3D-constructed scaffold, three-dimensional (3D) printing technology has been shown to be a promising method due to its various abilities, including the creation of complex constructs and their precise placement as well as a wide permissible range for materials. For effective tissue engineering, a scaffold must be easy to handle, biocompatible, and biodegradable to help cells to adhere, function, migrate, and proliferate well and to prevent inflammation [15]. In addition, the scaffold should have similar mechanical properties, especially the Young’s modulus, as the original region into which it is to be implanted to avoid stress shielding and implantation failure [16,17]. Synthetic and natural polymers such as PLA, PCL, collagen, and fibrin have been used in adipose tissue engineering; however, mismatched mechanical properties or unmatched degradation rates have been thresholds to overcome [18,19]. Poly (L-lactide-co-ε-caprolactone) (PLCL) is a co-polymer synthesized with L-lactide and ε-caprolactone through ring-opening polymerization. The mechanical properties of the PLCL co-polymer can be controlled by changing the monomer component [20]. It has flexible, stretchable, and elastic properties and degrades slowly, which means that it not only has more suitable properties than other synthetic polymers for soft tissue engineering, but also overcomes the limitations of native hydrogels and provides a three-dimensional spatial structure [21].

In regeneration for large defects, an efficient blood supply is absolutely necessary to deliver oxygen and nutrients and to remove waste products. Fat injection can be a good solution for a small defect; however, 3D-vascularized engineered tissue is a necessary challenge for the regeneration of large-sized adipose tissue with implantable constructs [22]. Thick implants, especially the central part, require vascularization to be used in vivo [1]; moreover, O_2_ is diffused less than 100–200 μm through tissue [23]. There are various strategies to enhance vascularization in tissue development, such as the pre-vascularization technique, growth factor-based technique, cell-based technique, and scaffold- and material-based techniques. Medical cost, initial angiogenesis, and functionality of the newly formed blood vessels have been considered as important conditions for the recovery of a critical size defect. Various strategies have been tried to develop vascularized tissue; however, they still have problems in forming functional vascularized tissues or organs [24]. Most cells are generally found within 100–200 μm of the nearest capillary for survival, and the maximum distance between capillaries is 200 μm. For these reasons, a highly developed neo-vascularized system of large- and micro-size blood vessels is required for tissue engineering [25,26,27,28,29].

The extracellular matrix (ECM) is a widely used material for scaffolds because it is composed of functional proteins, collagen, fibronectin, glycosaminoglycans (GAGs) and proteoglycans, which means that it is a favorable condition, providing a suitable microenvironment for cells and tissues to maintain their morphology and to differentiate [30]. The decellularization procedure is the protocol that removes the cellular components from the organs or tissues while maintaining the ECM components [31]. A complex mixture of decellularized ECM (dECM) is usually made into mixed hydrogels with collagen, hyaluronic acid, and fibroin for enhanced tissue regeneration [32]. In this regard, adipose tissue-derived dECM (adECM) is a potential material for adipose tissue regeneration because it has been reported that the adECM promotes regeneration and repair by providing a supportive microenvironment for adipogenic differentiation of induced cells [6,33] and by enhancing angiogenesis because it has growth factors related to neovascularization, such as VEGF [7,34].

The aim of this study was to develop a vascularized adipose tissue with a PLCL/dECM scaffold with the capability of cell recruitment and growth by providing the proper mechanical properties and microenvironment. The main focus of this study was to prepare a construct using the dual nozzle 3D printing technique with an elastic synthetic polymer and dECM hydrogel that induces angiogenesis and forms adipose tissue.

## 2. Results and Discussion

### 2.1. PLCL Characterization

**Figure 1:**
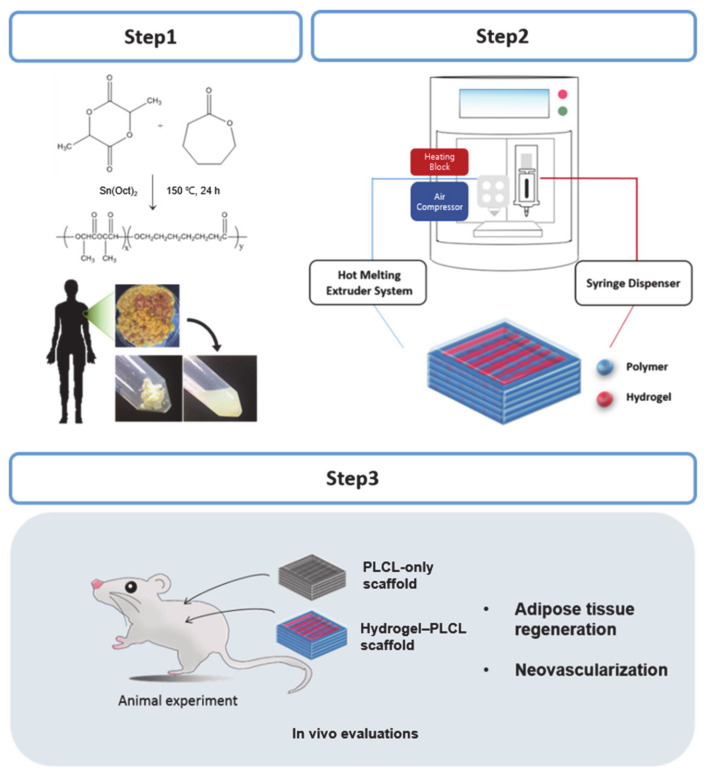
Schematic explanation of the research strategy. An elastic copolymer, PLCL, was synthesized by ring-opening polymerization, and the hydrogel was made with decellularized adipose tissue extracellular matrix (ECM) and collagen. Three-dimensional (3D) printing was performed with the PLCL and hydrogel inks with a hot melting extruder and syringe, respectively. The 3D-printed scaffolds were subcutaneously implanted in rats for the in vivo experiments.

**Figure 2:**
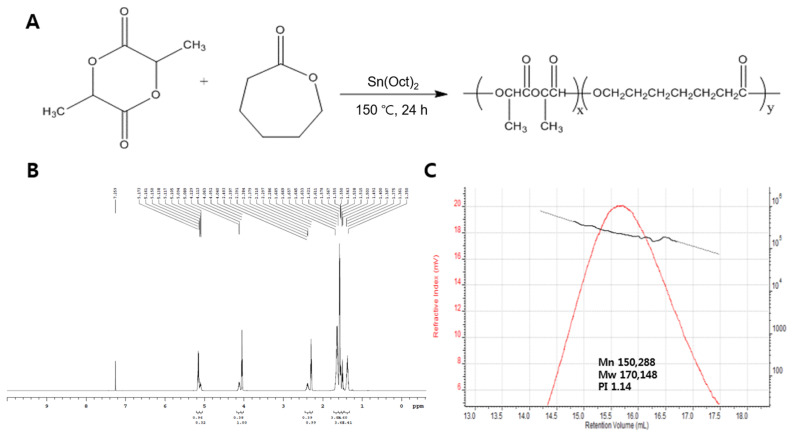
PLCL synthesis and characterization. (**A**) Scheme for the PLCL copolymer synthesis. (**B**) 600 MHz ^1^H-NMR spectrum of the PLCL copolymer (50:50). (**C**) Gel permeation chromatography (GPC) curve of the PLCL copolymer.

For adipose tissue engineering, a flexible and bioactive scaffold was fabricated, as shown in Figure 1. The PLCL co-polymer was synthesized by the ring-opening polymerization of L-lactide and ε-caprolactone in the presence of Sn(Oct)2 with continuous stirring under bulk conditions. A high conversion rate was expected under these conditions after a predetermined optimum reaction time. The polymerizations were carried out at 150 °C with a constant molar feed ratio (ƒL/ƒC) of 5:5. The distribution ratio of the PLCL was calculated by taking the ratio between the integration area of the peaks of the lactyl (Quartet, 5.160 ppm) and caproyl (Triplet, 4.051 ppm) units by 600 Hz ^1^H-NMR (Figure 2b). The molar ratio of L-lactide and ε-caprolactone in the co-polymer was 4.8:5.2. The overall yield of the PLCL co-polymer was 70%. Mn (number-average), Mw (weight-average), and molecular weight distributions (polydispersity, PDI = Mw/Mn) were determined by gel permeation chromatography (GPC) (Figure 2c). It was revealed that the Mn, Mw, and PDI of the PLCL co-polymer were 1.50 × 10^5^, 1.70 × 10^5^, and 1.14, respectively [20].

### 2.2. Characterization of dECM Hydrogel for 3D Priting Ink

Various scaffolds, including the extracellular matrix (ECM), have been used for diverse tissue or organ regeneration applications and medical strategies. The ECM produced by the resident cells of each tissue and organ can serve as an ideal biomimetic microenvironment for target cells which can accelerate tissue remodeling and healing in many organs [35,36,37,38,39]. Adipose tissue-derived ECM induces the migration and adipogenic differentiation of cells to form fat [40].

**Figure 3:**
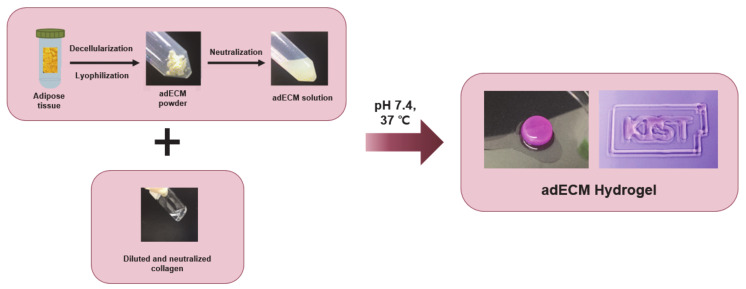
Preparation scheme for the mixed hydrogel using adipose tissue-derived decellularized ECM (adECM) and collagen. Human adipose tissue was decellularized by various processes and lyophilized. The resulting adECM was crushed into a powder and made into a hydrogel ink with collagen type I for 3D printing. Gelation and 3D printing of the hydrogel with various compositions of adECM and collagen was performed to optimize the printing condition. Bottom picture shows the optimized hydrogel (adECM:Col = 1:3).

**Figure 4:**
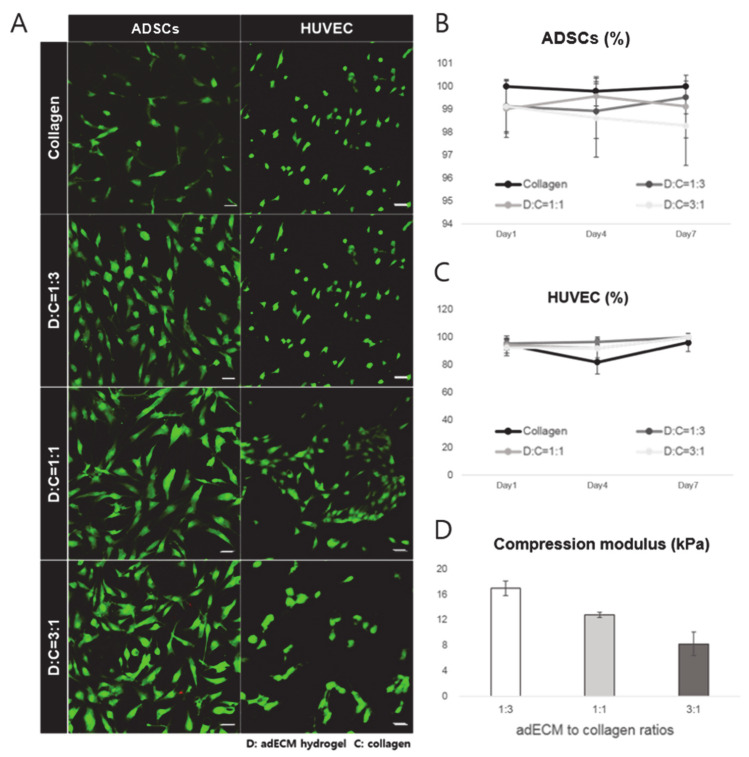
(**A**) LIVE/DEAD assay. Cell viability test was performed to optimize the hydrogel composition. Adipose-derived stem cells (ADSCs) and human umbilical vein endothelial cells (HUVECs) were seeded onto each of the hydrogels. The confocal images above were obtained after a 7-day incubation of cells. Green and red fluorescence represents live and dead cells, respectively. Scale bar = 50 μM. (**B**) Cell viability results of ADSCs. (**C**) Cell viability results of HUVEC. (**D**) Compression modulus of each hydrogel. All samples were evaluated in triplicate, and the error bar indicates the SD.

To promote adipogenic differentiation and angiogenesis, adipose tissue-derived decellularized extracellular matrix-based hydrogels were used to make a biocompatible ink. To preserve the residual ECM and maximize the cellular material loss [41], we used a modified method for adipose tissue with the physical, chemical, and enzymatic processes described elsewhere resulting in the successful decellularization of the tissues [42]. The successfully decellularized adECM powder was made into an ink with collagen type I for 3D printing (Figure 3). The adECM powder was dissolved with 10 mg of pepsin in 3% (*v*/*v*) acetic acid solution and neutralized with 10× Minimum Essential Media (MEM) and 5 N NaOH solution to adjust pH. The collagen was diluted to 3 mg/mL and neutralized with 10× MEM media, distilled water, and 1 N NaOH solution. Finally, the 0.3% collagen and 3% adECM neutralized solutions were mixed well and incubated at 37 °C for 30 min for gelation. Different adECM-to-collagen mass ratios were used, ranging from 1:3, 1:1, and 3:1 to determine the suitability of the ink in terms of printability and cell viability. Cell viability results (Figure 4) showed that all composition ratios of the mixed hydrogel had no cell toxicity and provided a proper environment for both adipose-derived stem cells (ADSCs) and human umbilical vein endothelial cells (HUVECs), which was evident by the more than 90% cell viability in each hydrogel. The collagen only, 1:1-adECM:collagen, and 1:3-adECM:collagen had better cell viability results for ADSCs in that order, although there was some difference, whereas the 1:3, 1:1, and 3:1-adECM:collagen had better cell viability results for the HUVECs (Figure S1). The compressive moduli of the 1:3-adECM:collagen, 1:1-adECM:collagen, and 3:1-adECM:collagen were 16.95, 12.80, and 8.20 kPa, respectively. In the printability test, the 1:3-adECM:collagen had the clearest printability compared to the others; thus, the 1:3-adECM:collagen hydrogel was selected as the ink for fabrication of the 3D scaffold. The 3:1-adECM:collagen hydrogel tended to be too runny and would spread during printing, so it was excluded from the printing test.

### 2.3. Characterization of the PLCL Scaffolds and the Hydrogel–PLCL Constructs

Porosity and pore size are significant factors for tissue regeneration. Proper scaffolds should have an excess of 50% porosity with at least a pore size of 100 μM [43,44,45]. Porosity and pore size are directly related to their functionality for tissue engineering applications. Moreover, openly porous and interconnected pores are essential conditions for providing oxygen, nutrients, and cell migration, as well as proliferation for vascularization and tissue formation. Generally, macropores are necessary to have enough space for vascularization, cellular infiltration, tissue ingrowth, and waste product removal [46,47,48,49,50,51].

Thus, the cellular behavior for new tissue reconstruction can be affected by the material properties of the scaffold such as the porosity, pore size, and interconnectivity [52]. Angiogenesis means the growth of new blood vessels from vasculature existing originally; thus, it is very important to supply oxygen and nutrients for tissue accumulation and wound healing during long-term tissue regeneration [53]. Larger pore sizes of approximately 160 to 270 μM facilitate angiogenesis throughout a scaffold, and many studies have shown that average pore sizes larger than 300 μM provide a better condition for vascularization [54,55,56].

**Figure 5:**
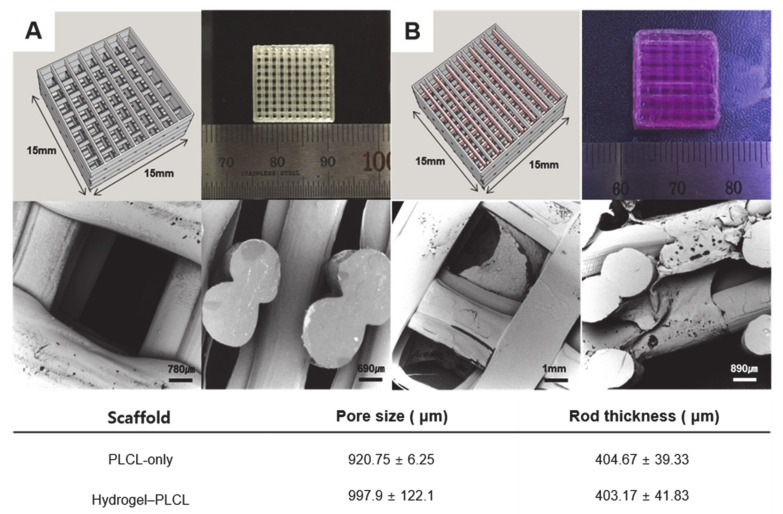
3D-printed scaffold. Standard Triangle Language (STL) file image for 3D printing, macroscopic image, and SEM images of the (**A**) PLCL-only scaffold and (**B**) Hydrogel–PLCL complex construct. The pore size and rod thickness were measured.

Figure 5 shows the Standard Triangle Language (STL) image files and printed scaffolds by the 3D printer (In vivo premium, Rokit, Republic of Korea) for the PLCL-only scaffold and hydrogel–PLCL construct, respectively. Each scaffold was printed quite accurately when compared to the bird’s-eye view images of the STL files as intended. The PLCL scaffolds were very flexible and stretchable, as shown in Figure S2. The morphology of the PLCL-only scaffolds and the hydrogel–PLCL constructs were observed by scanning electron microscopy (SEM). The open pores were highly interconnected and created surfaces on both the outside and inside of the scaffolds. The interconnected pore channels in the PLCL-only or hydrogel–PLCL scaffolds had a generally uniformed pore size of 920.75 ± 6.25 and 997.5 ± 122.1, while the mean rod thicknesses of the scaffolds were 404.47 ± 39.33 and 403.17 ± 41.83, respectively.

**Figure 6:**
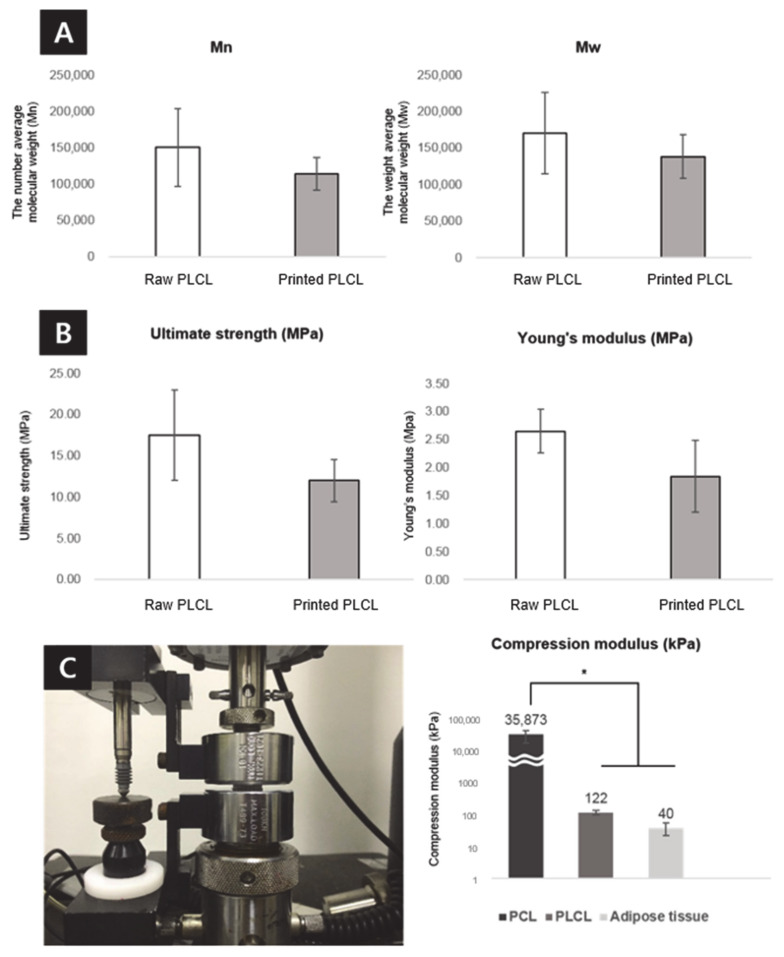
Evaluation of the molecular weight changes and mechanical properties. (**A**) The number (Mn, left) and weight (Mw, right) average molecular weight changes of the PLCL copolymer after 3D printing. Analysis of the mechanical properties of the PLCL copolymer before and after 3D printing, including the (**B**) ultimate strength (left) and Young’s modulus (right). (**C**) Compressive modulus value of the PCL, PLCL scaffolds and adipose tissue was evaluate. All samples were evaluated in triplicate, and the error bar indicates SD (* *p* < 0.01).

To evaluate the ultimate tensile strength change of the PLCL polymer before and after 3D printing, the ultimate strength and its Young’s modulus were determined and plotted as a graph (Figure 6). The PLCL copolymer is degraded by heat [57,58], which could be confirmed by molecular weight changes and Young’s modulus loss after 3D printing. The number (Mn) and average molecular weight (Mw) decreased from 150 kD to 114 kD and from 170 kD to 139 kD. The ultimate strength and Young’s modulus decreased from 17.50 ± 5.43 MPa to 11.99 ± 2.52 MPa, and from 2.65 ± 0.39 MPa to 1.85 ± 0.64 MPa. The compressive moduli of the PCL and PLCL were measured to confirm that their physical properties matched those of human adipose tissue, to avoid stress shielding and implantation failure. The compressive moduli of the scaffolds were approximately 3.6 MPa for the PCL, 122 kPa for the PLCL, and 40 kPa for adipose tissue. Tissue engineering scaffolds are required to have both synthetic materials with adjustable mechanical properties and natural materials with biomimetic properties. Synthetic polymers with a well-defined structure and without immunological problems such as PLA, PCL, and PLGA are widely used as a biomedical scaffold which has a crucial role in tissue engineering [59]. Among them, PCL is a representative material of tissue engineering [59,60,61]. PCL scaffolds used to be fabricated for soft tissue engineering [62,63]; however, as shown in this result, PCL has a very high modulus compared to native tissue. Meanwhile, in the case of the PLCL scaffold, it has a modulus value much closer to adipose tissue than that of the PCL scaffold. This result demonstrates that PLCL has a physical property that is more similar to adipose tissue, which is beneficial for regeneration, compared to the PCL.

### 2.4. In Vivo Studies

**Figure 7:**
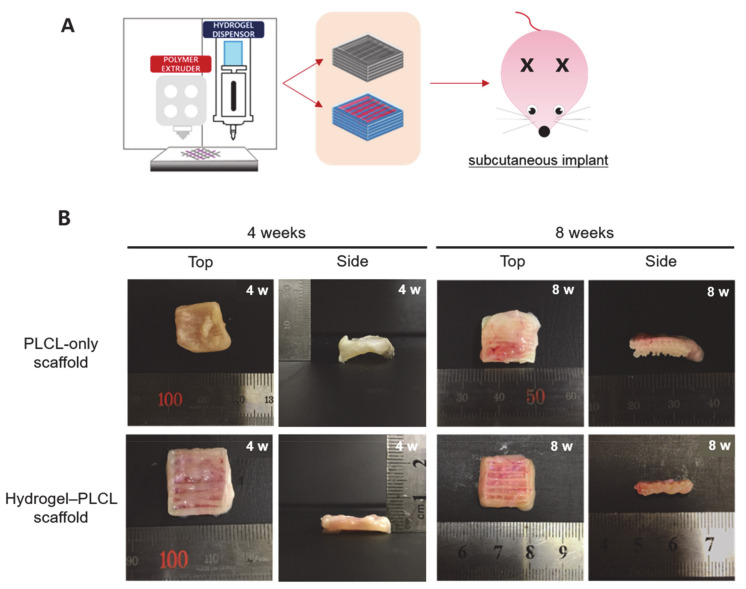
In vivo experiment. (**A**) Schematic image of the in vivo experiments. (**B**) Macroscopic images of the PLCL-only scaffolds and hydrogel–PLCL complex constructs after 4 and 8 weeks, respectively.

All animals for the in vivo experiments were treated in accordance with the standard operating protocols of the Institutional Animal Care and Use Committee at the Korea Institute of Science and Technology (KIST). All protocols were approved by the Institutional Review Board of Animal Experiments at KIST (Approval Number Kist-2019-009). Four and eight weeks after subcutaneous implantation, the scaffolds were harvested from the rats. Figure 7 shows the macroscopic view of the implanted scaffolds by each time point and group. Plenty more blood vessels were observed in the hydrogel–PLCL scaffold group than in the PLCL-only group.

**Figure 8:**
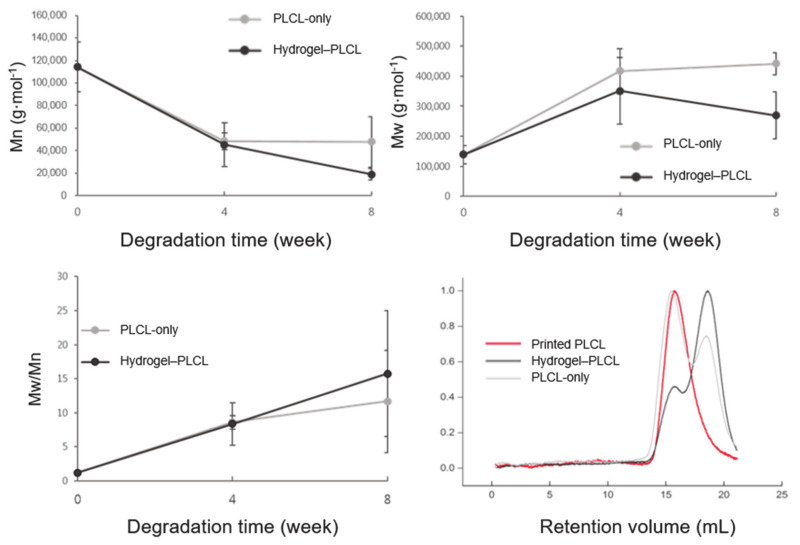
The number (Mn), average molecular weight (Mw), and polydispersity (PDI) value changes of the scaffolds from the in vivo degradation study.

Evaluation for the molecular weight change of the PLCL polymer in vivo was performed for eight weeks. The results show that the tendency for the changes in the number (Mn) and average molecular weights (Mw) gradually decreased or increased over the entire experiment period. The PLCL-only scaffolds had Mn decreases of 57.58% and 58.12%, and Mw increases of 200.67% and 219.03% at four and eight weeks after the subcutaneous implants, respectively. The hydrogel–PLCL constructs had larger Mn decreases of 60.37% and 83.41% and Mw increases of 153.48% and 94.46% at four and eight weeks, respectively (Figure 8). The result of increasing Mw can be interpreted as the short chains were unstable and degraded rapidly compared to long chains. The polydispersity index (Mw/Mn) increased gradually up to eight weeks post-implantation, indicating cleaved chains from the polymer. The PLCL has both crystalline and amorphous phases; the amorphous phase is more easily attacked by water compared to the crystalline regions [64]. Therefore, the changes in the average molecular weight of both the scaffolds could be explained by the following reason: the amorphous phases composed of CL, which is degraded faster than the crystalline regions composed of LA, were degraded in an earlier stage in the body.

### 2.5. Histological Analysis with Hematoxylin and Eosin (H&E) and Masson’s Trichrome (MT) Staining

**Figure 9:**
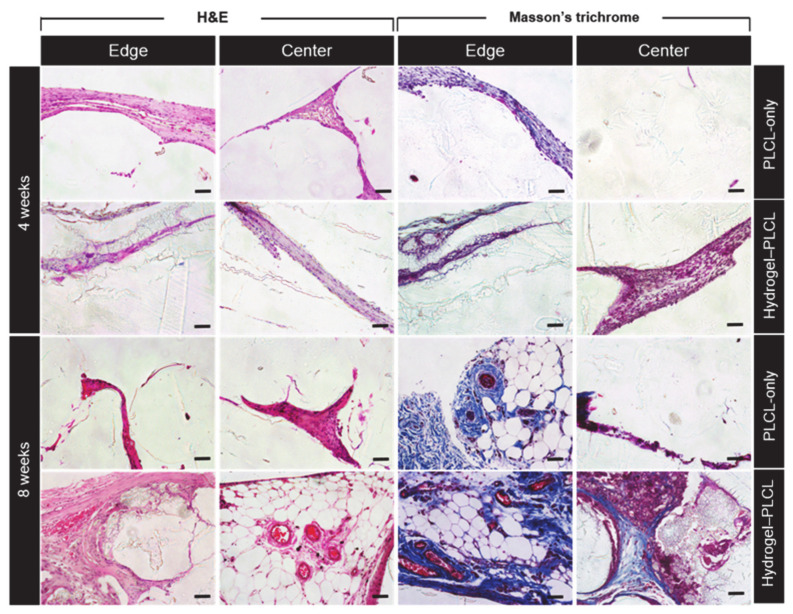
Histological analysis of the scaffolds after specific time points. The specimens were stained with hematoxylin and eosin (H&E) and Masson’s trichrome (MT). Scale bar = 50 μm.

For qualitative analysis, explanted scaffolds were made into 5 μM slides and stained with hematoxylin and eosin (H&E) and Masson’s Trichrome (MT) for histological analysis. Figure 9 shows the results of the staining of each group for each time point. As shown in the figure, in the fourth week, both groups had little fat accumulation and tissue formation. However, the difference is that tissue formation only occurred on the outside edge in the PLCL-only group, whereas the tissue was coming into and moving out of the scaffold in the hydrogel–PLCL group. In addition, fat accumulation and tissue formation were observed more in the center of the hydrogel–PLCL scaffold compared to the PLCL-only group, which means the hydrogel degradation behavior enhanced tissue formation [65,66]. Red blood cells and small blood vessels were observed in both groups. More significant differences between the two groups were observed at week 8. In the case of the PLCL-only group, a large amount of fat accumulation was observed on the outside of the scaffolds. Red blood cells, veins, and arteries were also found, but not in the center of the scaffold. There was no significant tissue or vessel formation in the center at eight weeks compared to four weeks. On the other hand, in the hydrogel–PLCL group, there were obvious differences such as the accumulation of fat, tissue formation, and vascularization. There was much tissue and fat coming into the scaffold as well as moving out; especially, there was plenty of adipose tissue with blood vessels in the center of the scaffold. A number of mature veins and arteries were found outside and inside of the scaffold, as well as lymphatic vessels. Lymphatic vessels are crucial for the recirculation of tissue fluid balance and cells that enter tissues from blood vessels [67]. An amount of sulfated GAG deposition was also observed in the hydrogel–PLCL group by the blue color of Masson’s trichrome, and it was found that the blood vessels, GAG, and tissue were formed between the scaffold both outside and inside of the scaffold. These results show that the hydrogel–PLCL constructs enhanced the tissue formation and promoted angiogenesis and vascularization.

### 2.6. Macrophage Infiltration Analysis

**Figure 10:**
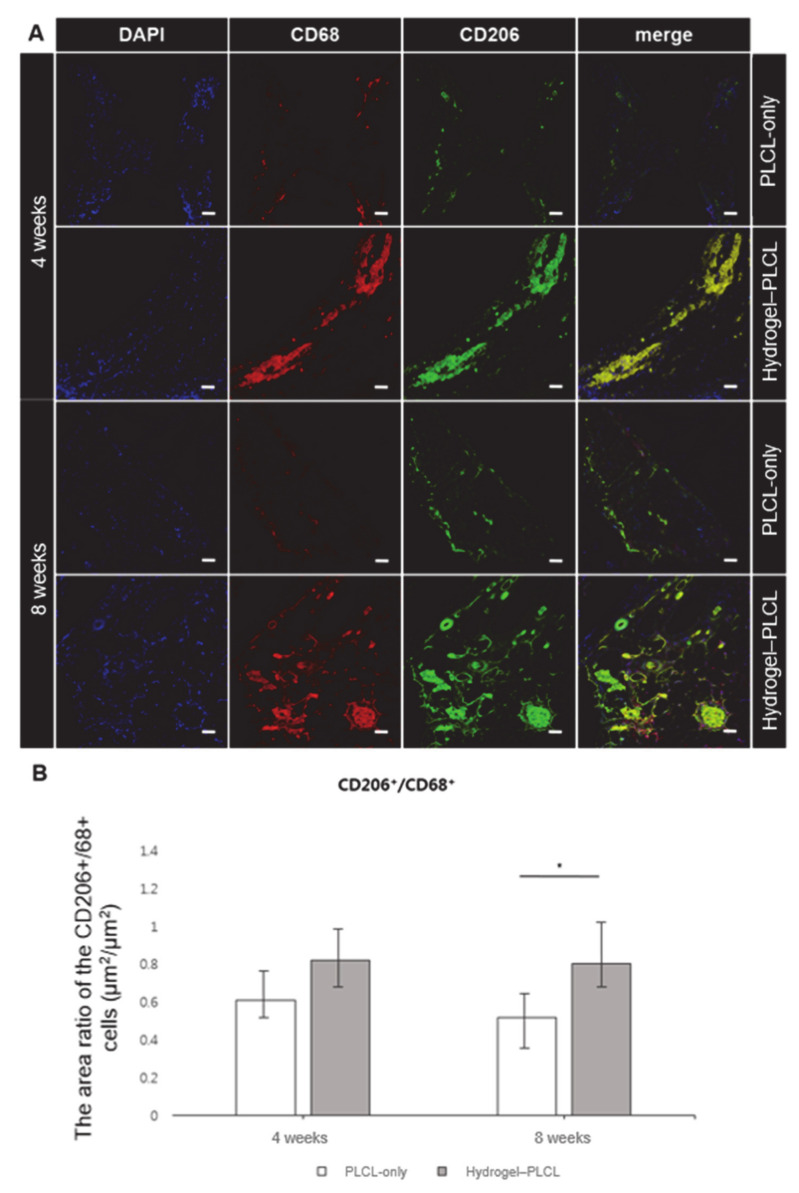
(**A**) Immunofluorescence images stained with CD68 and CD206 for investigation of macrophage infiltration. The images were obtained by confocal microscopy after 4 and 8 weeks in vivo (* *p* < 0.01). Scale bar = 50 μm. (**B**) Quantitative analysis of the macrophage infiltration area for identification of the M1 and M2 macrophages.

Immunofluorescence staining with CD68 and CD206 was performed to investigate macrophage infiltration, as shown in Figure 10a. CD68 and CD206 were stained with a red and green color, respectively. In addition, DAPI was stained as a blue color. CD68 is a pan-monocyte/macrophage marker, and CD206 is an M2 macrophage marker. Macrophages are an important factor to detect early inflammatory responses in tissue engineering [68,69]. Macrophage differentiation is affected by the cytokine environment. M1 macrophages produce the pro-inflammatory cytokines, phagocytize microbes, and induce an immune response. These factors predominantly have an effect in inflammation, tissue injuries, and apoptosis [70,71,72]. Activated M2 macrophages are alternatively associated with tissue remodeling and angiogenesis by downregulating inflammation and eliminating tissue wastes and apoptotic bodies. Additionally, they also produce polyamines to induce collagen accumulation [73,74,75,76]. Significant amounts of CD68+ and CD206+ cells were found in the hydrogel–PLCL constructs. For quantitative analysis, the macrophage infiltration area (μm^2^) for the CD68+ and CD206+ cells were calculated to identify the M1 and M2 macrophages, and the area ratio of CD206+/CD68+ cells (μm^2^/μm^2^) was also calculated. The calculated values were plotted as a graph (Figure 10b). The area ratio of the CD206+/68+ cells (μm^2^/μm^2^) for the PLCL-only group was 0.61 ± 0.29 and 0.52 ± 0.16 for four weeks and eight weeks, respectively, and that of the hydrogel–PLCL group was 0.82 ± 0.21 and 0.80 ± 0.16, respectively. Intriguingly, the ratio of the CD206+/CD68+ cells was significantly higher in the hydrogel–PLCL group compared with that in the PLCL-only group, which might reflect the macrophages in the former moving toward the tissue regeneration state [76,77].

### 2.7. Analysis of Angiogenesis and Vascularization in the Scaffolds

**Figure 11:**
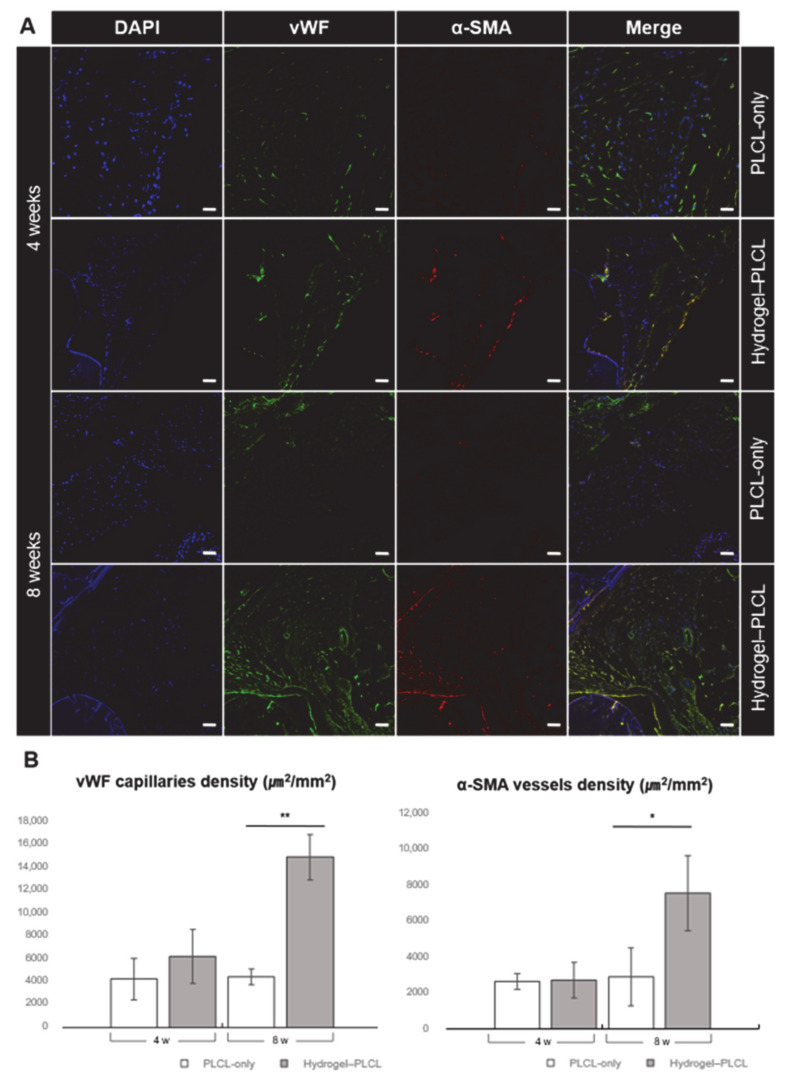
Analysis of vessel formation in the specimens after 4 and 8 weeks in vivo. (**A**) Immunofluorescence image of the PLCL-only group and hydrogel–PLCL complex construct 4 and 8 weeks after subcutaneous implantation. (**B**) von Willebrand factor (vWF) and α-smooth muscle actin (α-SMA) density of the specimens 4 and 8 weeks after subcutaneous implantation (stained area μm^2^/total area mm^2^) (* *p* < 0.01, ** *p* < 0.0001). Scale bar = 50 μm.

To confirm the endothelial lineage differentiation in the hydrogel–PLCL construct, the expression of von Willebrand factor (vWF) and α-smooth muscle actin (α-SMA) was studied by immunofluorescent staining at four and eight weeks after implantation. vWF and α-SMA are well-known specific endothelial markers. vWF is used to detect panendothelial cells, and α-SMA targets pericytes and mature vessels surrounded with actin-positive vascular smooth cells. vWF staining is observed for both immature and mature vessels; however, α-SMA is observed for mature vessels [78,79,80,81]. The confocal image in Figure 11 shows that the formation of matured blood vessels gradually increased in the hydrogel–PLCL group from the fourth to the eighth week. Early angiogenesis at four weeks occurred in both the PLCL-only scaffolds and hydrogel–PLCL constructs. vWF and α-SMA densities of each scaffold were analyzed by immunofluorescence staining. A few small, immature vessels were detected. However, there was no significant formation of mature capillaries in the PLCL-only group at eight weeks, while matured capillaries did form in the hydrogel–PLCL constructs (Figure S3). The density of the capillaries increased from 4218.7 ± 1787.1 μm^2^ to 4400.1 ± 696.8 μm^2^, and 6156.6 ± 2357.4 μm^2^ to 14736.3 ± 1955.0 μm^2^ for the PLCL-only scaffolds and the hydrogel–PLCL constructs, respectively. Moreover, the arterial density detected by α-SMA staining was also higher in the hydrogel–PLCL constructs at 7603.2 ± 2109.4 μm^2^ compared to the PLCL-only scaffolds at 2913.7 ± 1620.6 μm^2^. The immunofluorescent staining showed that the adECM hydrogel in the hydrogel–PLCL constructs promoted the recruitment of endothelial cells and their differentiation.

### 2.8. Quantitative Analysis by Real-Time Polymerase Chain Reaction

**Figure 12:**
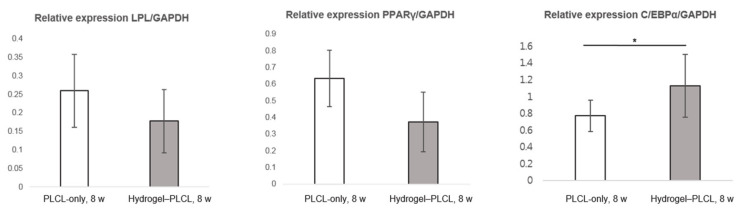
Analysis of quantitative real-time polymerase chain reaction results. Relative mRNA expression of LPL, PPARγ and C/EBPα in the PLCL-only scaffolds and the hydrogel–PLCL complex constructs (* *p* < 0.01).

Figure 12 shows the gene expression data at 8 weeks for both scaffolds. The PLCL-only scaffold had a higher gene expression for peroxisome proliferator-activated receptor γ (PPARγ); however, there was no significant statistical difference between the groups. In contrast, C/EBPα was expressed much higher in the hydrogel–PLCL construct group compared to the PLCL-only group. The relative gene expression analysis of lipoprotein lipase (LPL), PPARγ, glyceraldehyde-3-phosphate dehydrogenase (GAPDH), and CCAAT/enhancer binding protein β (C/EBPα) was performed at eight weeks after implantation. Markers related to adipogenic differentiation were identified at the mRNA level, and the expression levels of all genes were normalized relative to that of the GAPDH level. Lipoprotein lipase (LPL) is a very early marker of adipogenic differentiation induced by cell–cell contact. Peroxisome proliferator-activated receptor γ (PPARγ) is mediated by CCAAT/enhancer binding protein β (C/EBPβ), and C/EBPα is also mediated by C/EBPβ. PPARγ is cross-regulated with C/EBPβ, but C/EBPα cooperates with PPARγ, inducing the transcription of adipocyte genes which leads to the creation and maintenance of the adipocyte phenotype. C/EBPα is known as a terminal differentiation marker and works in a synergistic manner with PPARγ. Therefore, LPL is expressed at a very early stage of adipogenic differentiation, and PPARγ is gradually expressed in the intermediate stage; then, C/EBPα has a critical role in the late stage of differentiation [82]. As shown by the results in this study, the PLCL-only group was in the intermediate stage of adipogenic differentiation. Furthermore, it means that the hydrogel–PLCL construct group was favorable for the promotion of adipose tissue regeneration by leading to full adipogenic lineage differentiation.

## 3. Materials and Methods

### 3.1. Materials

L-Lactide (Purac Biochem, Gorinchem, Netherlands) and ε-Caprolactone (Sigma Aldrich, St. Louis, MO, USA) were used as received. As an initiator, 1-dodecanol (Sigma Aldrich, St. Louis, MO, USA) was used, and tin (II) 2-ethylhexanoate (Sigma Aldrich, St. Louis, MO, USA) was used as a catalyst. All chemicals and solvents were of analytical grade and were used as received.

### 3.2. Synthesis and Characterization of PLCL

PLCL co-polymer was synthesized by the ring-opening polymerization of L-lactide (100 mmol) and ε-caprolactone (100 mmol), which was catalyzed by Sn(Oct)2, as described elsewhere [20,83]. Briefly, the reaction mixture containing the monomers, catalyst and initiator diluted in dried toluene was poured into a 50 mL glass ampoule. The ampoule was completely sealed under a vacuum after purging three times with nitrogen and then polymerized at 150 °C for 24 h (Figure 2a). After the reaction, the product was dissolved in chloroform and filtered through an 8 μm pore filter paper. Then, the polymer was precipitated into an excess of methanol, filtered, and dried under a vacuum over 3 days.

To identify the distribution ratio of the PLCL copolymer, nuclear magnetic resonance (NMR) spectroscopy was performed on a 600 MHz ^1^H-NMR spectrometer (Agilent, Santa Clara, CA, USA) with deuterated chloroform (CDCl_3_, Aldrich, St. Louis, MO, USA) solutions. Spectra were obtained with 1% (*w*/*v*) solutions in chloroform with 0.03% (*v*/*v*) tetramethylsilane as the standard. The distribution ratio was calculated from the NMR spectra. The number and weight of the molecular weights were determined using gel permeation chromatography (GPC, Viscotek TDA 302, Malvern Instruments Ltd, Worcestershire, United Kingdom) calibrated with polystyrene. Chloroform was used as a mobile phase at a flow rate of 1.0 mL/min.

### 3.3. Preparation of the Decellularized Extracellular Matrix (dECM)-Based Ink

The decellularization process for adipose tissue was performed by following the method published elsewhere with a small modification [42]. Adipose tissue was obtained from Seoul National University. The adipose tissue was chopped into fine pieces first and decellularized in 0.5% sodium dodecyl sulfate (SDS) solution for 48 h. The 0.5% SDS solution was replaced every 12 h. After that, the tissue was removed from the 0.5% SDS solution and treated with isopropyl alcohol to eliminate the lipids for 48 h, changing the solution every 12 h. The decellularized and dilapidated tissues were then washed with PBS and treated with a solution of 0.1% peracetic acid in 4% ethanol for 4 h. Finally, the tissues were washed with PBS for 72 h and lyophilized. The lyophilized tissues were crushed into a fine powder with a cryomiller (FreezerMill, SPEX Sample Prep., Metuchen, NJ, USA). The adECM powder was stored in a −80 °C freezer until the gelation protocol.

The adECM powder was sterilized with a supercritical carbon dioxide system at 200 bar for 1 h [84]. The sterilized adECM powder was taken as required and dissolved with 10 mg of pepsin (P7125, Sigma, St. Louis, MO, USA) in 3% (*v*/*v*) acetic acid (Sigma, St. Louis, MO, USA) solution using a mixing rotator at 4 °C for 48 h. The adECM solution had an acidic pH, so it was neutralized with sterile 10× MEM (11430-030, Gibco, Waltham, MA, USA) and sterile 5 N NaOH solution to adjust the pH.

Rat tail collagen I (354236, Corning, St. Louis, MO, USA) was diluted to 3 mg/mL and neutralized with sterile 10× MEM media, sterile distilled water, and sterile 1 N NaOH solution, while the temperature was kept below 10 °C to avoid gelation. After adding the calculated volume of all components which were neutralized separately, the 0.3% collagen and 3% adECM solutions were mixed well. To optimize the biological and mechanical conditions, adECM and collagen solutions were mixed at 3:1, 1:1, and 1:3 (*v*/*v*) ratios.

The mechanical properties of the hydrogels with different adECM-to-collagen ratios were measured by unconfined compression tests (*n* = 3). A cylinder mold with a 6 mm diameter was prepared to make regular-shaped hydrogels. The mixtures of the hydrogels were poured into the mold. After allowing 30 min for gelation, uniformed cylindrical hydrogels were finally prepared and compressed using a 10 kN load cell at the rate of 1 mm/min (Instron 5900, Norwood, MA, USA). The compressive modulus was determined as the force per unit area applied to the hydrogels during compression and calculated as the slope of the initial straight line in the stress–strain curve.

The cell viability measurements for the various adECM and collagen ratios were performed with live/dead cell viability assay kits (L3224, Thermo Fisher scientific, Waltham, MA, USA) (*n* = 3). First, 200 μL of the mixed hydrogels were poured into a 96-well plate and incubated at 37 °C in a humidified atmosphere containing 5% (*v*/*v*) CO_2_ for 30 min for gelation. ADSCs and HUVECs were then seeded onto each of the hydrogels (1.5 × 10^4^ cells/well). Later, on days 1, 4 and 7, Calcein AM 5 μL and Ethidium homodimer (EthD-1, 20 μL) were mixed in 10 mL of PBS and spread onto the mixed hydrogel. Calcein AM (green)- and EthD-1 (red)-stained live and dead cells, respectively. The stained cells were observed and counted manually using confocal microscopy to calculate the percentage of live and dead cells (Zeiss 520, Jena, Germany).

### 3.4. Fabrication of the PLCL Scaffolds and the Hydrogel–PLCL Constructs

Figure 1 shows a schematic illustration of all steps for our experiments. The PLCL-only scaffolds and hydrogel–PLCL scaffolds were fabricated with a dual-nozzle 3D printer (In vivo premium, Rokit, Seoul, Republic of Korea). The PLCL was extruded from a hot-melting extruder nozzle using a pneumatic air compressor with high pressure for the PLCL, which is very elastic [85].

The hydrogel–PLCL constructs were fabricated with the dual-nozzle system. The hydrogel solution containing the collagen and adECM was printed with a 10 mL syringe while maintaining temperatures below 10 °C to avoid gelation before printing. The PLCL was printed with the same method that was used for the PLCL-only scaffold. The hydrogel solution and PLCL were printed alternately at 37 °C in a humidified atmosphere condition to prevent the hydrogel solution from drying out before gelation. Both the PLCL-only and hydrogel–PLCL complex scaffolds were placed under a UV light for 15 min for sterilization before the in vivo experiments.

### 3.5. Scanning Electron Microscopy (SEM) Analysis

The surface morphology of the constructs was examined by scanning electron microscopy (SEM) (Phenom-world, Eindhoven, Netherlands). The top surface and cross-section of the samples were observed. The samples were coated for 60 s with gold using a sputter-coater (Bal-tec SCD 005, Baltac prä parathion, Niesgrau, Germany).

### 3.6. Mechanical Tests

The mechanical properties of the PLCL scaffold were measured by unconfined compression tests (*n* = 3). Uniformed scaffolds with a constant shape and size were fabricated with the PLCL co-polymer and PCL polymer. Human adipose tissue was cut into a cylindrical shape with a stamping mold to compare the compression modulus with the PCL and PLCL scaffolds. Adipose tissue and the PCL and PLCL scaffolds were prepared and compressed using a 10 kN load cell at a rate of 1 mm/min (Instron 5900, Instron®, Norwood, MA, USA). The compressive modulus was determined as the force per unit area applied to the hydrogels during compression and calculated as the slope of the initial straight line in the stress–strain curve.

To evaluate the ultimate tensile strength, raw PLCL (before printing) and printed PLCL were made into thin films. The films were mounted on a universal testing machine and subjected to tensile testing at a crosshead speed of 1 mm/min. Means and standard deviations were expressed in MPa, and the Young’s modulus was calculated as the slope of the initial straight line in the stress–strain curve.

### 3.7. In Vivo Experiments

SD rats (7-week-old, male; Dae-han biolink co., Ltd, the Republic of Korea) were randomly allocated into two groups: PLCL-only group (*n* = 4) and PLCL with hydrogel (adECM:collagen = 1:3) group (*n* = 4). All equipment for the surgery was sterilized with an autoclave in advance. Sterilized constructs were individually implanted into the bilateral dorsal subcutis pouches of the rats to evaluate the ability of adipose tissue regeneration and vascularization. The rats were temporarily anesthetized with ethyl ether. Gentamicin (Gibco, Waltham, MA, USA) was used as antibiotics (dose, 80 mg kg^−1^) after surgery. The constructs were harvested at 4 and 8 weeks after implantation.

The molecular weight changes during the in vivo study were analyzed by collecting samples after specific time points. The samples were chopped and dissolved in CHCl_3_ and then purified in methanol. The resulting samples were dried for 3 days at 60 °C, after which the samples were dissolved in CHCl_3_ to evaluate their molecular weight.

### 3.8. Evaluation of Adipose Tissue Regeneration with Histological Analysis

The constructs were fixed in 10% (*v*/*v*) buffered formalin (HT110116, Sigma, St. Louis, MO, USA) for 24 h, dehydrated in a graded ethanol series, embedded, and then made into paraffin blocks for histological analysis. Each sample was sectioned by a microtome at a 5 μm thickness. The sectioned samples (*n* = 4) were stained with hematoxylin and eosin (H&E) and Masson’s trichrome (MT) to examine the tissue morphology and collagen composition. The samples were examined under light microscopy (Nikon, Tokyo, Japan) [86].

### 3.9. Macrophage and Angiogenesis Assessment of the Constructs

To evaluate the immune response by macrophage infiltration 4 and 8 weeks after implantation, the sample section was blocked and incubated with mouse monoclonal anti-cluster of differentiation 68 (CD 68, 1:100, ab955, Abcam, Cambridge, United Kingdom) for both M1 and M2 macrophages, and CD 206 (1:100, SC-34577, Santa Cruz Biotechnology, Inc., Dallas, TX, USA) for M2 macrophages, overnight.

To examine angiogenesis, rabbit anti-human von Willebrand factor antibody (vWF, 1:400, ab6994, Abcam, Cambridge, United Kingdom) and monoclonal mouse anti-human α-smooth muscle actin antibody (α-SMA, 1:200, ab7817, Abcam, Cambridge, United Kingdom) were used as primary antibodies to stain endothelial cells (ECs) and vascular smooth muscle cells (SMCs). After an overnight incubation at 4 °C, the samples were incubated with Alexa Fluor 488 goat anti-rabbit IgG (1:1000, A11034, Life Technologies, Carlsbad, CA, USA) and Alexa Fluor 594 goat Anti-mouse IgG (1:1000, A11062, Life Technologies, Carlsbad, CA, USA) for 1 h at RT. Cell nuclei were observed by staining with 4’, 6-diamidino-2-phenylindole (DAPI, Molecular Probes) for up to 5 min. Slides were observed under a confocal microscope (Zeiss 520, Jena, Germany) [83,84,86,87]. The area (μm^2^) of the DAPI+, CD68+, and CD206 staining was measured with the Image J program to estimate the immune response in the scaffolds by the ratio of macrophages per whole cell. The value of the CD206+/CD68+ indicates the ratio of the M2 macrophages/all macrophages in the scaffolds which enhance angiogenesis and tissue regeneration.

### 3.10. Adipose Tissue mRNA Expression Analysis by RT-PCR

The RNA of the cells from each of the samples was isolated and purified using the RNeasy® mini kit (Qiagen, Hilden, Germany), following the manufacturer’s instructions, to quantify the gene expression. RNA extraction was measured using Nanodrop (Nanodrop® ND-1000, Thermo Fisher Scientific, Waltham, MA, USA), after which reverse transcription for RNA was performed with the Omniscript® System (Qiagen, Hilden, Germany). RNA samples were incubated in a dry bath (WiseTherm® HB-R, DAIHAN Scientific, Seoul, Republic of Korea) at 37 °C for 60 min. The real-time PCR was performed with the Power SYBR® Green PCR Master Mix (Applied Biosystems, Foster City, CA, USA) using the 7500 Real-Time PCR system (ABI prism 7500, Applied Biosystems, Foster City, CA, USA). The oligonucleotide primers for lipoprotein lipase (LPL), peroxisome proliferator-activated receptor gamma (PPARγ), glyceraldehyde-3-phosphate dehydrogenase (GAPDH) and CCAAT/enhancer binding protein alpha (C/EBPα) were designed based on references or published gene sequences (NCBI) [88,89]. GAPDH primers were used for the control genes, and the other genes were normalized by GAPDH. Relative gene expression levels were analyzed using the 2^−ΔΔCT^ method.

### 3.11. Statistical Analysis

The samples were assessed at least in triplicate, and all results are expressed as the mean ± standard deviation values. All statistical analyses were performed by Student’s *t*-tests, and statistical significance was set at * *p* < 0.01 or ** *p* < 0.0001 for all results.

## 4. Conclusions

We have developed adipose tissue-derived dECM hydrogel based PLCL constructs to develop vascularized adipose tissue. The 3D printing technique was used for scaffold fabrication, which could fabricate patient-specific scaffold for later clinical application. It was confirmed that the highly elastic PLCL co-polymer used in this study has more approximate physical properties as native adipose tissue compared to the other polymers. Furthermore, we revealed that adECM hydrogel promotes adipose tissue reconstruction by encouraging neovascularization and tissue formation. A high level of PPARγ gene expression was observed in the PLCL-only group; however, histological and immunological staining analysis revealed that the tissue formation and vessel formation were markedly poor compared to that of the hydrogel–PLCL construct group. Indeed, C/EBPα, the late-stage marker, was expressed much more in the hydrogel–PLCL construct group, with significantly different values. Our results demonstrated that the hydrogel–PLCL constructs promoted efficient adipogenic differentiation with a developed vascularized structure. Consequently, we have demonstrated the feasibility of a therapeutic method for large-sized adipose tissue regeneration by developing vascularized tissue with the 3D-printed hydrogel–PLCL scaffold. Therefore, it could be a good alternative for adipose tissue engineering, and we expect that it is applicable to not only adipose tissue but also to other soft tissue regeneration.

## Data Availability

All data are reported in the manuscript and in the supplementary information.

## Supplementary Materials

The following are available online at https://www.mdpi.com/1422-0067/22/6/2886/s1, Figure S1: Cell viability test was performed to optimize the hydrogel composition. The confocal images above were obtained after a 7-day incubation of ADSCs and HUVECs. All samples were evaluated in triplicate, and the error bar indicates the SD. Scale bar = 50 μm; Figure S2: Flexible and stretchable test for the 3D-printed PLCL scaffold. The scaffold bent easily and recovered to its original shape after printing; Figure S3: Confocal images of the in vivo experiments for immune response: DNA, vWF, a-SMA, and merged images at 4 and 8 weeks after subcutaneous implantation; 8w, 20× confocal. Scale bar = 50 μm; Video S1: Flexibility test for the 3D-printed PLCL scaffold; Video S2: Stretchability test for the 3D-printed PLCL scaffold.
