# Supplementary material for: Enhanced Regeneration of Vascularized Adipose Tissue with Dual 3D-Printed Elastic Polymer/dECM Hydrogel Complex
Source: Int J Mol Sci. 2021 Mar 12;22(6):2886. doi: 10.3390/ijms22062886 (PMC7999751; doi:10.3390/ijms22062886)
Supplement: Supplementary file 1 [file ijms-22-02886-s001.zip › Supplement Information_IJMS_1.docx]

Supporting Information

for

**Enhanced Regeneration of Vascularized Adipose Tissue with Dual 3D printed Elastic Polymer/dECM hydrogel complex**

Soojin Lee ^1,2^, Hyun Su Lee ^2^, Justin J. Chung ^1^, Soo Hyun Kim ^1,3^, Jong Woong Park ^4^, Kangwon Lee ^5,*^, Youngmee Jung ^1,6,*^

^1^ Center for Biomaterials, Biomedical Research Institute, Korea Institute of Science and Technology, Seoul; 02792, Republic of Korea; dltnwls830@snu.ac.kr (S.L.); chungjj@kist.re.kr (J.J.C.); soohkim@kist.re.kr (S.H.K.)

^2^ Program in Nanoscience and Technology, Graduate School of Convergence Science and Technology, Seoul National University, Seoul; 08826, Republic of Korea; hyun118soo@snu.ac.kr (H.S.L.)

^3^ NBIT, KU-KIST Graduate School of Converging Science and Technology, Korea University, Seoul; 02841, Republic of Korea

^4^ Department of Orthopedic Surgery, Korea University Anam Hospital, Seoul; 02841, Republic of Korea; ospark@korea.ac.kr (J.W.P.)

^5^ Department of Applied Bioengineering, Graduate School of Convergence Science and Technology, Seoul National University, Seoul; 08826, Republic of Korea

^6^ School of Electrical and Electronic Engineering, YU-KIST Institute, Yonsei University, Seoul; 03722, Republic of Korea

* Correspondence: kangwonlee@snu.ac.kr (K.L.); winnie97@kist.re.kr (Y.J.)


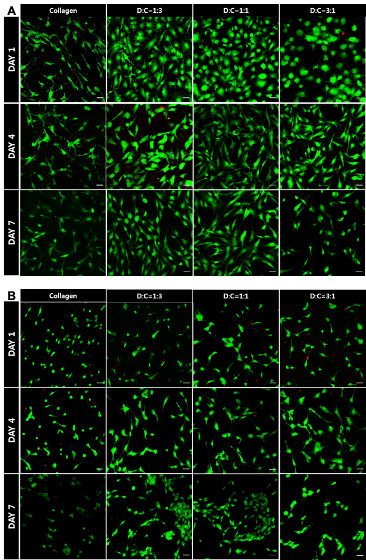


**Figure S1.** Cell viability test was performed to optimize the hydrogel composition. The confocal images above were obtained after a 7-day incubation of ADSCs and HUVECs. All samples were evaluated in triplicate, and the error bar indicates the SD. Scale bar=50 μm.


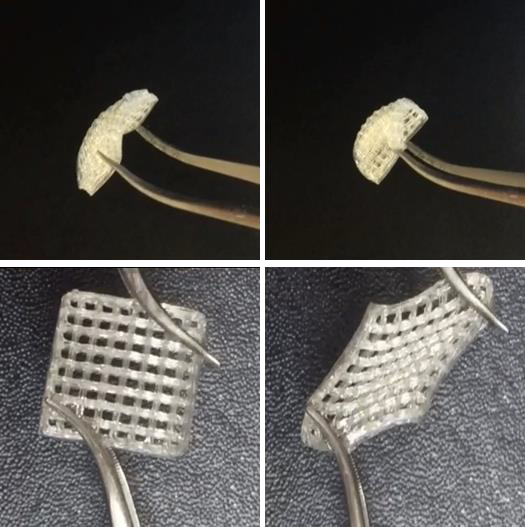


**Figure S2.** Flexible and stretchable test for the 3D printed PLCL scaffold. The scaffold bent easily and recovered to its original shape after printing.

**
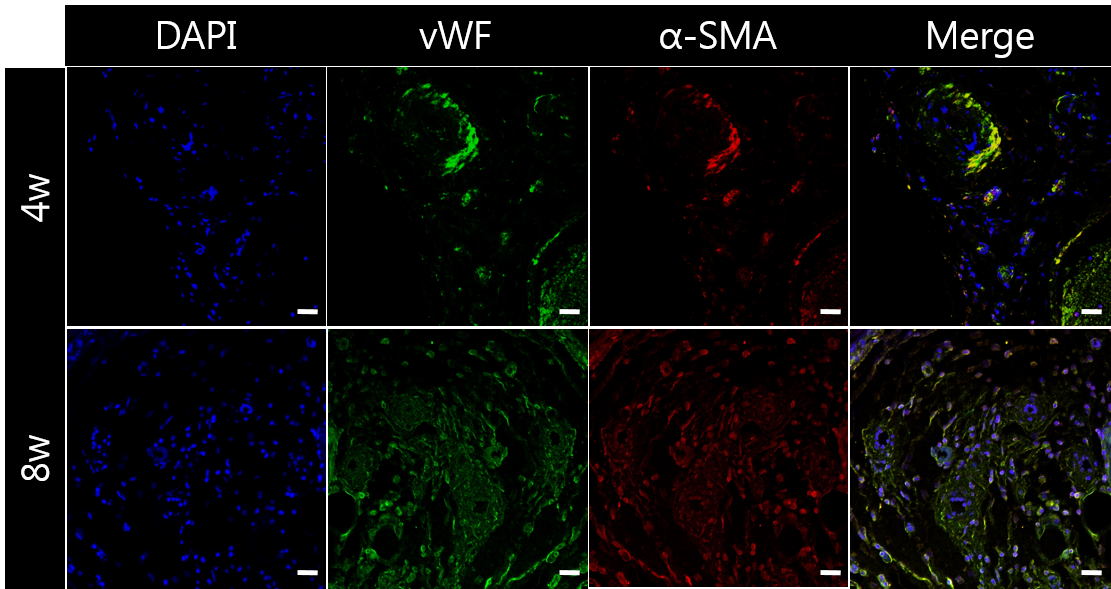
**

**Figure S3.** Confocal images of the *in vivo* experiments for immune response: DNA, vWF, a-SMA, and merged images at 4 and 8 weeks after subcutaneous implantation. 8w, 20x confocal. Scale bar=50 μm.
